# A novel approach to sclerotomy closure in pars plana vitrectomy: a pilot study

**DOI:** 10.1186/s40942-022-00414-z

**Published:** 2022-09-11

**Authors:** Mallory K. Suarez, Rebecca M. Sappington, Bartlett Hayes

**Affiliations:** 1 Dept. Ophthalmology, Wake Forest School of Medicine, Winston-Salem, NC 27157 USA; 2 Dept. Neurobiology and Anatomy, Wake Forest School of Medicine, Winston-Salem, NC 27157 USA

**Keywords:** Scleral wound closure, Pars plana vitrectomy, PPV, Platelet-rich plasma, PRP, Polyethylene glycol (PEG) sealant

## Abstract

**Background:**

Methods of sclerotomy closure following a vitrectomy, including the use of sutures, have been associated with complications such as inflammation, foreign body sensation, and infection. Here, we test an innovative approach to scleral wound closure following pars plana vitrectomy that involves plugging the wound. We investigated several materials with the intent of using products that were either already approved by the FDA for other types of procedures or were biocompatible patient-derived materials.

**Methods:**

We examined whether scleral wounds could be sealed by a clot or internal “plug” rather than a suture or an external adhesive. We tested patient-derived materials (platelet-rich plasma (PRP) and whole blood) as well as polyethylene glycol (PEG) sealant. Whole blood and PRP were prevented from clotting prematurely using sodium citrate, and were clotted for the study with thrombin. Polyethylene glycol (PEG) sealant was prepared according to manufacturer’s recommendations. We used fresh-frozen cadaveric porcine eyes. We tested several methods to form plugs using the above materials, as well as various methods to deliver the plugs into the sclerotomy incisions. We used a novel technique of manual vitrectomy. Successfully generated and implanted clots were tested for efficacy with the Seidel test.

**Results:**

Polyethylene glycol (PEG) sealant fractured during our attempts at molding and inserting the plug. In contrast, both whole blood and PRP yielded successful plugs for insertion. We molded a whole blood clot plug by allowing it to clot inside a 20-gauge angiocath catheter and we successfully delivered it through a 23G trocar. At baseline, no wound leakage was apparent. However, the whole blood clot dislodged during the Seidel test. We successfully molded and delivered a PRP clot plug using a tapered 2-20 μl pipette tip, using MAXGrip Forceps to push it through into the wound. No scleral wound leakage was noted at our baseline physiologic infusion pressure. Furthermore, the PRP clot plug prevented scleral wound leakage up to a pressure of 60 mmHg and was confirmed with the Seidel test.

**Conclusion:**

Our findings suggest that insertion of a clot plug made from either whole blood or PRP may be an effective strategy for scleral wound closure following pars plana vitrectomy. Further testing in preclinical models is warranted to further refine the materials and methods, since this appears to have the potential to improve the closure of the scleral wounds after pars plana vitrectomy.

## Background

Previous methods of sclerotomy closure following a vitrectomy, including the use of sutures, have proven to have many associated complications including inflammation, foreign body sensation, secondary infection, suture breakage, and astigmatism [1]. Ocular adhesives have been used as an alternative to sutures in certain ocular procedures, although with some materials there are concerns regarding biocompatibility, biomechanical characteristics different from native tissue, potential for toxicity, reliable and rapid application, efficient sealing of the wound, and permeability to gas and nutrients [1]. Current ocular adhesives can be organized into two main classes: synthetic adhesives which include cyanoacrylate-based and linear polyethylene glycol (PEG)-based adhesives and naturally-derived polymers which include protein-based and polysaccharide-based adhesives [1, 2].

### Synthetic adhesives

Cyanoacrylate-based adhesives have been shown to result in ocular surface irritation and discomfort, and defective sclerotomy wound closure leading to sclerotomy leakage [3]. Polyethylene glycol (PEG) sealant (Ocular Therapeutix, Inc., Bedford, MA, USA) is a Polyethylene Glycol based ocular adhesive that is FDA-approved and is used for sealing corneal incisions [1]. Polyethylene glycol (PEG) hydrogel polymers are biocompatible, safe and constructed to have varying flexibility and absorption [4]. Polyethylene glycol (PEG) sealant comes prepackaged in three parts: a N-hydroxysuccinimide (NHS)-terminated 4-arm PEG prepolymer, a tri-lysine amine crosslinker, and a diluent dropper. The components are mixed to form a hydrogel in under 30 s [1]. Polyethylene glycol (PEG) sealant is composed primarily of water (89% by weight) and stays on ocular surfaces for 1–3 days until it hydrolyzes, softens, and is cleared through tear fluid. When applied to a cataract incision, it can withstand intraocular pressures ranging from 11 to 29 mmHg [1]. According to the FDA Summary of Safety and Effectiveness Data, adverse effects include hypotony, inflammation, corneal edema, or allergic reactions.

### Naturally-derived polymer adhesives

Previous in vivo studies indicate that fibrin and fibrinogen-based adhesives mimic the biological process of clot formation, showing confirmed re-absorbability, biodegradability, and biocompatibility [1]. The therapeutic efficacy of fibrin glues is attributed to its slow polymerization which allows more time for intraoperative placement and naturally-derived polymers are biologic and do not produce toxic metabolites [5]. In a study comparing fibrin glue to cyanoacrylate tissue adhesive, the fibrin glue demonstrated accelerated healing with less corneal vascularization but required more time for adhesive formation [6]. The use of fibrin glue is not limited to external surfaces, and therefore is an advantageous material for use under the conjunctiva and amniotic membranes [5]. However, natural-based polymers carry some risk of virus transmission as some of the natural-based adhesives do [1]. The use of fibrin adhesives may also be limited by low adhesive and tensile strength [7].

### Alternative synthetic materials

Surgicel (Johnson and Johnson, Somerville, NJ, USA) and Gelfoam (Pharmacia & Upjohn Co., New York, NY, USA) are FDA approved absorbable hemostatic agents used in a variety of surgical procedures and have the potential to be utilized for sclerotomy closure [8]. Hemostatic agents such as Surgicel have shown efficacy for control of capillary, small arterial, and venous hemorrhage, and wound healing [9]. Gelfoam has been reported to form giant-cell granulomas at the site of implantation with the potential for bacterial growth. One case report cited the formation of a Surgicel granuloma, which occurred as the retained hemostatic material of Surgical can mimic abscesses and cause foreign body reactions [10].

### Alternative biological adhesives

Recent studies demonstrate the successful use of platelet rich plasma (PRP) in several ocular procedures including dormant ulcers, corneal perforations, dry eye, and ocular surface surgical reconstruction [11]. A PRP clot is prepared from the patient’s own blood and is advantageous to wound healing due to the presence of platelets and the extended release of growth factors which aid in wound healing [11]. A study by Babu et al. in [12] compared the use of PRP to that of an inverted internal limiting membrane flap for treatment of large macular holes and found that not only was the safety and efficacy of both approaches equivalent, but also patients treated with PRP did not have reported postoperative inflammation, hypotony, endophthalmitis or retinal detachments [12].

Several procedures exist for the preparation of PRP for use in ophthalmic procedures. A solid PRP clot can be obtained by collecting a patient's blood using 3.2% sodium citrate as an anticoagulant. The patient’s blood is then centrifuged for 10 min at 1600 rpm which separates the anticoagulated blood into different layers with platelet-poor plasma on top, PRP below, and white and red cells at the bottom [11]. After the PRP is extracted, it can be used to prepare a PRP clot by mixing the PRP with either thrombin or 10% calcium chloride [11]. PRP can also be prepared by drawing fresh blood from the patient and running it in the centrifuge before it coagulates. If the clot is prepared in this way, a piece which is an appropriate size and shape must be cut from the clotted plasma for use in the eye.

### Study rationale

In this study, we tested an innovative approach to scleral wound closure following pars plana vitrectomy that combines the methodological premise of fibrin-based adhesives with the biocompatibility advantages of patient-derived materials. We examined whether an internal “plug”, rather than an external adhesive seal, could be used for scleral wound closure. Although previous studies examine the use of clotted blood, clotted plasma, and polyethylene glycol (PEG) sealant for ophthalmic use, there are no reports researching these materials as “plugs'' or “clots” for scleral wound closure in pars plana vitrectomy. While Surgicel and Gelfoam are also potential alternative materials, we excluded these substrates based on the adverse effects and foreign body reactions previously reported for each when used in neurosurgical applications [9]. Our findings have the potential to modify and improve the current surgical approach to ocular wound closure from both technical and materials perspectives.

## Methods

### Tissue procurement and preparation

**Fig. 1:**
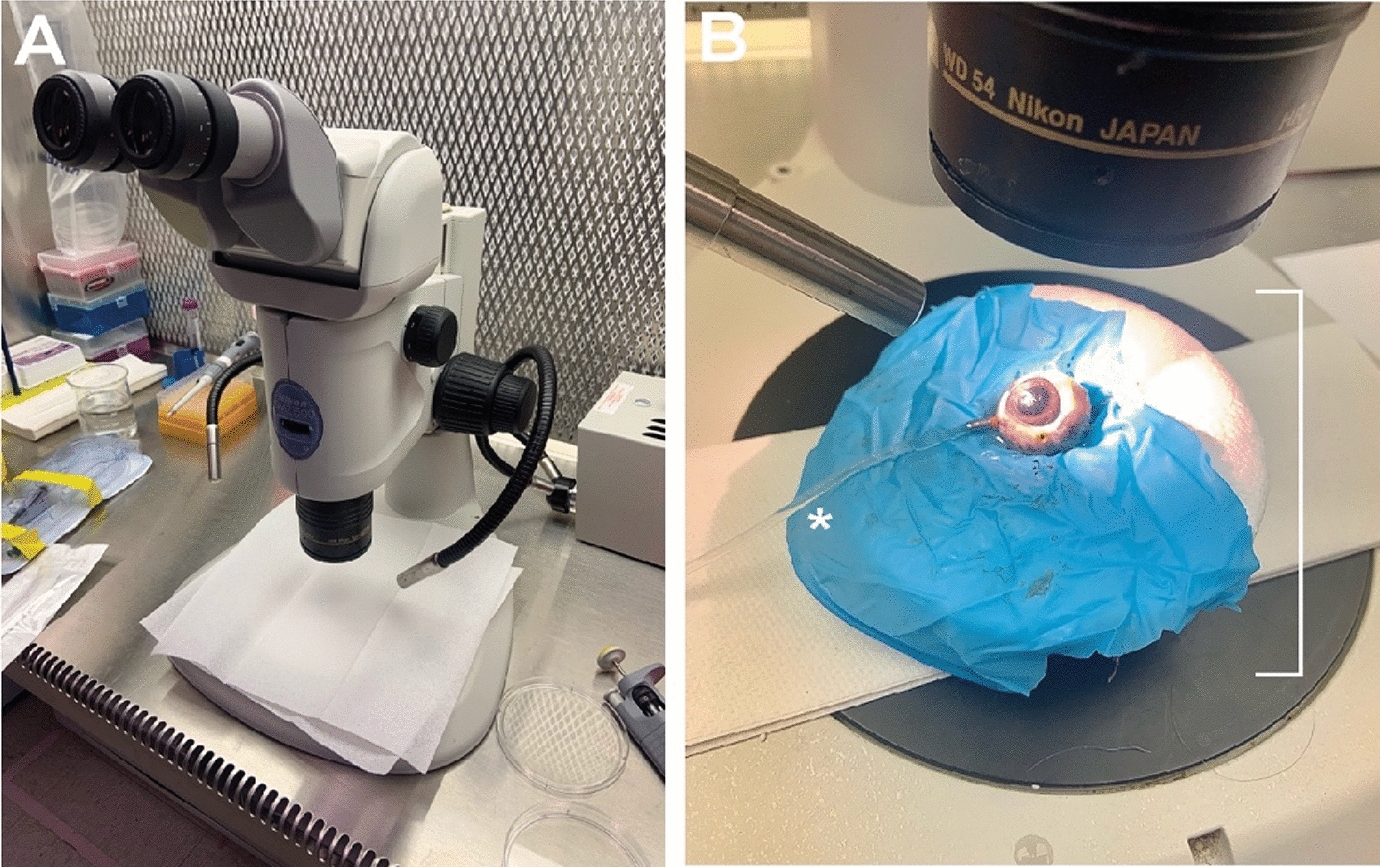
Porcine model of manual pars plana vitrectomy. **A** Display of experimental set-up with the dissecting microscope under the horizontal flow hood. **B** Porcine eye affixed to a Styrofoam mount

We obtained cadaveric Yorkshire porcine eyes (*n* = 3) as a gift from Dr. James Jordan, Department of Cardiothoracic Surgery, Wake Forest School of Medicine. Immediately upon enucleation, porcine eyes were placed in dry ice and stored in dry plastic laboratory test tubes in a −20 °C freezer. Within 48 h of enucleation and storage, porcine eyes were thawed for 30 min at room temperature. Eyes were affixed to a Styrofoam mount under a dissecting microscope (Nikon Instruments, Inc., Melville, NY) within a horizontal flow hood (Fig. 1). Remnants of the conjunctiva and tenon’s capsule were dissected off the sclera prior to vitrectomy.

### Manual vitrectomy of porcine eye

To perform this study, we needed to create a porcine model for scleral incisions in post-vitrectomized eye. Difficulties in accessing a veterinary research vitrectomy machine led us to develop a method for manual vitrectomy of a cadaveric porcine eye that does not require surgical equipment for mechanical pars plana vitrectomy. A 23-gauge trocar was used to place a trans-scleral cannula for the infusion line. The trocar was then removed for safety, but the wound was marked with a surgical marking pen so it could be located more easily after the manual vitrectomy. A #11 scalpel blade was utilized to make a transverse incision one quadrant away from the trocar wound and 4 mm from the limbus. The scalpel blade was inserted perpendicular to the ocular surface until the full width of the No. 11 blade had been reached. A 7–0 Vicryl suture was used to place an X-shaped suture, with two parallel passes across the wound. The suture was left loose, and the needle was removed for safety purposes to avoid accidental injury. With the suture in place, the vitreous was then manually removed by applying external pressure to the eye to squeeze the vitreous out of the 11-blade incision. A considerate amount of digital pressure must be applied to ensure that the vitreous is completely removed from the porcine eye.

The X-shaped suture was tightened and tied to secure the incision. The 23-gauge trocar cannula was re-inserted into the initial pre-placed sclerotomy, and an infusion line was connected to the cannula using 0.9% sodium chloride Injection USP, and this inflated the eye with physiologic intraocular pressure. To ensure the 11-blade incision was watertight, a Seidel test was performed at the incision site. Another 23-gauge trocar was used to place a second trans-scleral cannula 90 degrees from the first. A 23-gauge Grieshaber MAXGrip Forcep was utilized to open the valve of the trocar to ensure that it leaked, confirming that the vitreous was successfully removed. To test our materials for scleral wound closure, the 23-gauge trocar was removed, leaving a scleral wound which leaked saline infusion fluid, further confirming the adequacy of the vitrectomy.

### Platelet rich plasma (PRP) clot preparation

PRP was prepared in collaboration with the Hematology Lab at Atrium Health Wake Forest Baptist. PRP was prepared as previously described in a study by Alio et al. in 2015 with some modification [11]. Procedural modifications were instituted to improve accessibility to physicians during surgical procedures. Briefly, two vials (10 ml each) of whole blood were extracted using 3.2% sodium citrate anticoagulant. Whole blood was lightly centrifuged at 1600 rpm for 10 min for separation of platelet poor plasma, PRP, and blood cells. PRP was extracted from both vials and transferred to a single sterile vial. To create the PRP clot, a standard Denville Scientific Pipette was used to draw up 200 μL of platelet rich plasma, which was then placed into an endorphin tube. 100 μl of thrombin (Siemens Medical Solutions, Malvern, PA) was then added to the endorphin tube creating a 2:1 mixture of plasma and thrombin for clot formation. Once the thrombin was added to the plasma, clot activation occurred within 5 s, so methods piloted for mold preparation had to be executed as soon as the thrombin was inserted to the endorphin tube of PRP.

### Clotted whole blood preparation

Whole blood was prepared in collaboration with the Hematology Lab at Atrium Health Wake Forest Baptist. Two vials (10 ml each) of whole blood were extracted using 3.2% sodium citrate anticoagulant. For clotted blood preparation, we utilized thrombin as a clot activator, mixing three drops of approximately 20ul of thrombin (Siemens Medical Solutions, Malvern, PA) into a small vial of whole blood. As soon as thrombin was added to the vial of whole blood, the mixture was drawn into 16,18-, and 20-gauge angiocath tips on 1 ml Luer-Lok syringes and left to clot for 15 min.

### Polyethylene glycol (PEG) sealant clot preparation

Polyethylene glycol (PEG) sealant was obtained commercially and prepared according to manufacturer’s recommendations (Ocular Therapeutix, Inc., Bedford, MA, USA). Briefly, two drops of diluent were added to the white deposit and mixed until clear. This solution was then mixed with the blue deposit for 2–3 s. The solution was aspirated before it clotted, using a 20-gauge IV catheter angiocath on a Luer-Lok syringe. We allowed the PEG sealant to clot in the angiocath tips for 5 min.

## Results

Freshly enucleated cadaveric porcine eyes were promptly frozen to minus-80 degrees Celsius. Prior to the study a porcine eye was thawed at room temperature. We tried various methods for creating a plug, including PRP, whole blood, and PEG Sealant, for delivery into sclerotomy incisions for sclerotomy closure in 23-gauge pars plana vitrectomy. We tested several methods of clot delivery for each substrate to identify a technique that could improve wound. Successfully generated clots were tested for efficacy with gradual elevations in IOP up to 60 mmHg.

### Scleral wound closure with PRP

**Fig. 2:**
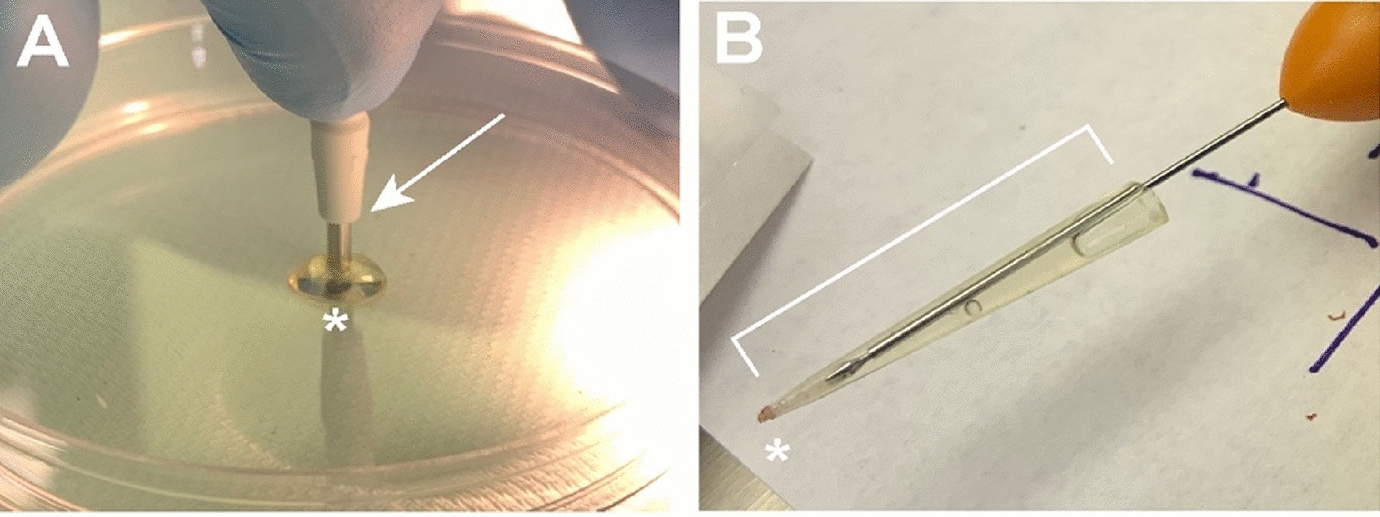
PRP clot formation by punch technique. **A** Cutting clotted PRP with a 2 mm dermatological punch. **B** Clotted PRP in a micropipette tip with forceps holding a piece of foam that was used as a plunger to push the PRP into the wound

Following clot activation, a 2 mm dermatological hole punch was utilized to obtain a 2 mm piece of clotted PRP (Fig. 2A). A pair of 23-gauge Grieshaber MAXGrip Forceps were used to deliver the clot through the 23-gauge trocar to plug the scleral wound as the trocar was removed. The consistency of the clotted PRP made it mechanically difficult to handle and deliver through the vitrectomy port without dislodging the clot. The clotted PRP could not be easily nor reliably inserted into the scleral wound through the 23-gauge trocar. After five trials, we deemed this method unsuccessful. As a result, we modified the methodology and attempted to further cut down the clotted PRP with micro scissors after molding it with the 2 mm dermatological hole punch, but the process was very tedious. Even with this modification of our technique we could not yield a reliable method of manually cutting the PRP down into a favorable cylindrical shaped clot to plug the scleral wound, therefore it was unsuccessful.

**Fig. 3:**
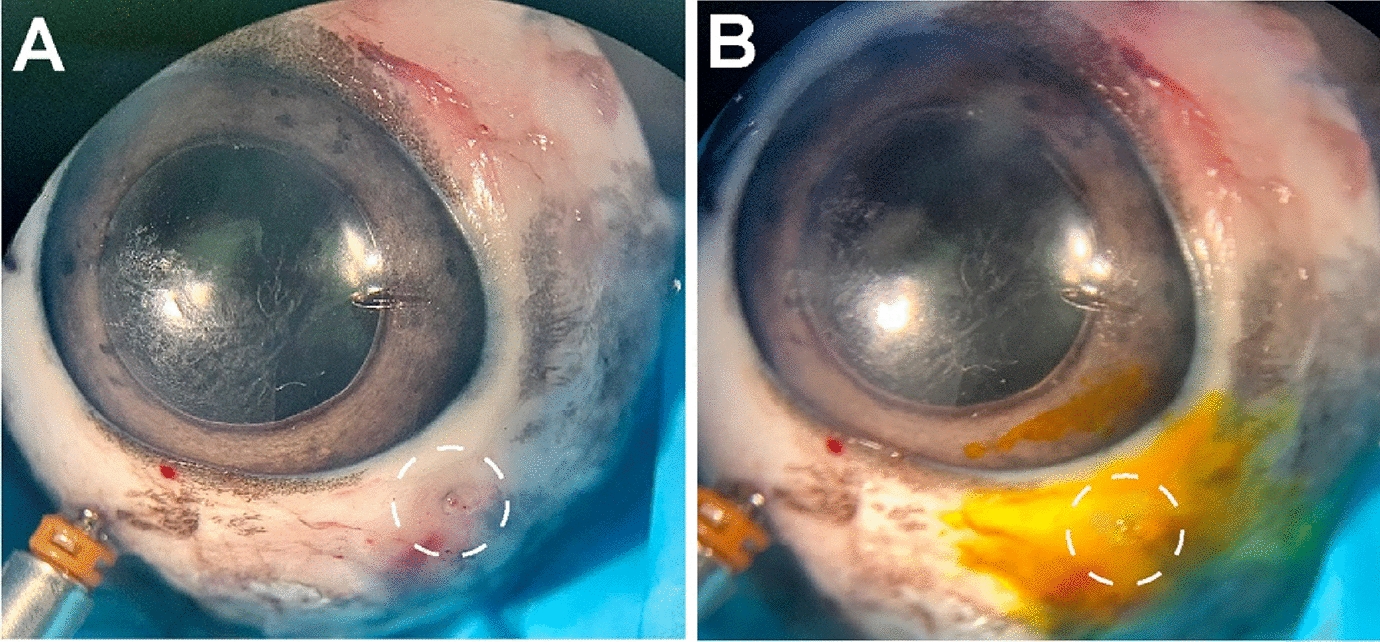
PRP clot insertion and testing. **A** Clotted PRP plug in the sclerotomy. **B** Performance of the Seidel test

As a second technique of clot preparation and delivery, we removed the 23-gauge trocar and placed a 2-20 μl pipette tip up against the scleral wound to deliver the clotted PRP into the wound (Fig. 2B). We created a plunger to push the clot by using a 2 mm diameter piece of foam held with a pair of 23-gauge Grieshaber MAXGrip Forceps. The micropipette tip was placed into the edge of the scleral wound, and the forceps with the foam plunger were used to push the clotted PRP into the scleral wound. We performed three trials using this methodology, the final trial successfully plugged the scleral wound (Fig. 3A). The Seidel test was then performed to test the effectiveness of the clotted PRP at increasing levels of intraocular pressure, and it was watertight up to 60 mmHg of intraocular pressure (Fig. 3B).

**Fig. 4:**
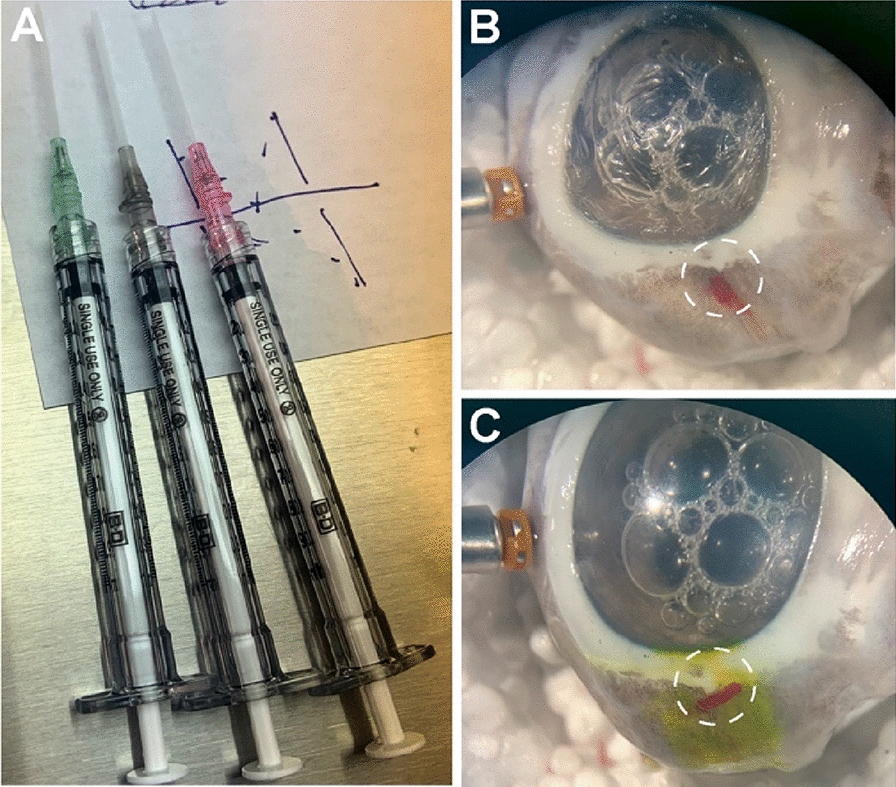
Angiocath method of clot formation and insertion. **A** 16, 18, and 20-gauge shielded IV catheter angiocath tips used as molds to create a cylindrically shaped clot of PRP and whole blood. **B** Clotted blood in the sclerotomy. **C** Performance of the Seidel test

In another method of clot preparation from PRP, we utilized 16, 18, and 20-gauge shielded IV catheter angiocath tips as molds to create a cylindrically shaped clot of PRP (Fig. 4A). The mixture successfully clotted within the original endorphin tube from which it was aspirated but failed to clot inside the angiocath tips. This methodology was repeated several times, and it was concluded that the PRP could not reliably clot inside of the angiocath tips. It remained a liquid consistency which could not be pushed through the angiocath tip into the 23-gauge trocar to plug the scleral wound, therefore this method was unsuccessful for clot preparation and delivery.

### Scleral wound closure with whole blood

Utilizing the same methodology that we used for PRP clot preparation in the angiocath tips (Fig. 4A), the IV angiocath catheter tip with the clotted whole blood inside, was cut with scissors into smaller 6-8 mm cylindrical pieces. After comparison of the different IV angiocath cylindrical clots, we determined that the cylindrical clotted molds from the 20-gauge IV angiocath could be delivered the most effectively through the 23-gauge trocar to plug the scleral wound. After three attempts of delivery of clotted whole blood using both the 16-gauge and 18-gauge angiocaths this technique of delivery proved unsuccessful.

The cylindrical piece of the 20G angiocath tip was aligned to the opening of the 23-gauge trocar and a 24-gauge IV angiocath tip was used to push the clot out through the 23-gauge trocar as it was being removed and into the scleral wound. After several attempts, the clotted whole blood was able to successfully plug the scleral wound in one trial (Fig. 4b). Unfortunately, the clot stuck to the fluorescein strip during the Seidel test, and the clot was dislodged.

### Scleral wound closure with polyethylene glycol (PEG) sealant

**Fig. 5:**
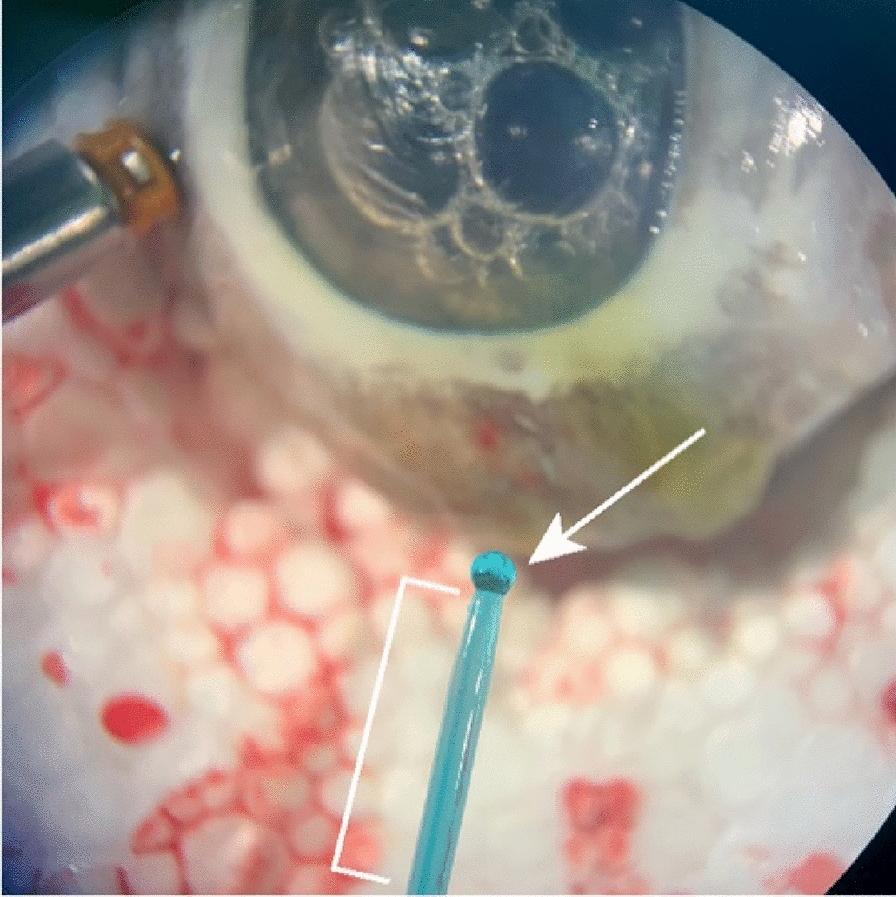
Angiocath method of polyethylene glycol (PEG) sealant clot formation. 20-gauge IV catheter angiocath tip used as a mold for PEG sealant

After clot formation, 20-gauge IV angiocath tips with PEG sealant were cut with scissors to obtain cylindrical PEG sealant clots (Fig. 5). The 20-gauge IV catheter cylindrical piece with clotted PEG sealant was placed at the edge of the opening of the 23-gauge trocar. More than five trials were performed in an attempt to push the PEG sealant clot through the trocar cannula. It was concluded that the PEG sealant adhesive yielded plugs which unfortunately were easily fragmented and could not successfully be used for plug formation. This innovative use of PEG sealant for the formation of a clot might require the addition of another component to give the clot more elasticity and make it less likely to fragment.

## Discussion

The objective of this study was to evaluate various materials as plugs for 23-gauge sclerotomy incisions as an innovative approach for scleral wound closure following 23-gauge pars plana vitrectomy. With a long-term goal of improving patient outcomes, our approach combined the methodological premise of fibrin-based adhesives with the biocompatibility advantages of patient-derived materials.

In this study, we successfully created a porcine model for closing scleral incisions by performing manual vitrectomies as outlined in our methods. In future studies that require porcine models for post-vitrectomized eyes, this methodology may be used in place of a veterinary vitrectomy instrument, which may not be as readily accessible. With each porcine eye utilized in this study, our methodology of manual vitrectomy allowed us to prepare porcine eyes which were confirmed to be successfully vitrectomized and reliable models for testing our materials for scleral wound closure.

Several trials across different methodologies of clot delivery yielded both successful and unsuccessful attempts at plugging scleral wounds. The initial technique of delivery of clotted PRP directly through the trocar cannula was unsuccessful, in part because intraocular forceps are not an optimal instrument for placing a plug in the scleral wound. In addition, the gelatinous consistency of clotted PRP made it very difficult to securely deliver directly through the trocar cannula without it dislodging or breaking apart. Even upon further reduction of clot size, the clot would dislodge from the forceps as it was passed through the valve in the trans-scleral cannula and could not successfully plug the wound.

In one trial the clotted PRP was successfully delivered through a 2-20 μl pipette tip into the eye, using a pair of 23G Grieshaber MAXGrip Forceps holding a 2 mm piece of foam sponge as a plunger and was stabilized without ocular wound leakage up to a pressure of 60 mmHg. The success of this technique is attributed to the funneled shape of the micropipette tip which was an efficient model for clot delivery. The smooth surface of the micropipette tip along with the funneled shape were advantageous properties which allowed for easy delivery of the clot directly into the scleral wound. The foam used as a plunger was also able to compress as it pushed the clot down the pipette. In future studies, surgical instruments that are approved for use intraoperatively with a similar shape and functionality to the micropipette tip should be tested as well.

In one trial with clotted whole blood, a clot prepared in a 20G IV angiocath tip was successfully delivered through the 23G trocar, plugging the scleral wound of the trocar with no ocular wound leakage at baseline. Clotted whole blood had a viscosity similar to the clotted PRP but did not have as much stability when molded for plug formation. With increasing intraocular pressure, it was dislodged by the fluorescein strip during the Seidel test. This may be attributed to the cylindrical shape of the clot molded from the 20G IV angiocath tip, which may not provide as much stability to the clot as intraocular pressure increases.

Trials utilizing PEG Sealant proved to be unsuccessful due to the properties of the PEG Sealant rather than the techniques of delivery. PEG Sealant does not have the stability to be utilized as a plug for wound closure. It is easily fragmented making it difficult to deliver into scleral wounds and does not have the stability to withstand increased intraocular pressure especially on exposure to wet surfaces, which would make it very difficult to use intraoperatively.

This study was a proof of concept that provided preliminary evidence that clotted PRP and clotted whole blood could be feasible options for the future of scleral wound closure. In our trials, PRP was proven to be more effective at preventing scleral wound leakage at higher intraocular pressures. Studies outlining the use of PRP for the treatment of ocular disorders have also given evidence of its safety and effectiveness. Further studies will be needed to continue to refine the methodology associated with clot plug formation, to standardize plug diameter, shape, and length, for its optimal use intraoperatively.

Furthermore, future well-controlled, randomized, and live animal studies will be required to determine the efficacy of this as an adjunct or alternative to other methods of treatment for scleral wound closure in pars plana vitrectomy.

## Conclusions

In this pilot study, we developed a manual vitrectomy method for cadaveric porcine eyes to test the efficacy of clotted PRP, clotted whole blood, and PEG Sealant to plug scleral wounds associated with 23-gauge pars plana vitrectomy. Our methodology of manual vitrectomy was used successfully on porcine eyes without the use of a veterinary vitrectomy machine. The success of the manual vitrectomy was further confirmed by creation of a scleral wound which leaked saline infusion fluid post-vitrectomy. The three materials tested displayed a range of wound closure efficacies. Clotted PRP placed directly into the wound using a micropipette and clotted whole blood placed directly into the wound using a 20-gauge angiocath catheter successfully plugged leaking scleral wounds at a physiologic intraocular pressure. A plug of clotted PRP remained stable up to an intraocular pressure of 60 mmHg without wound leakage, making it the most effective of the three materials we tested for plugging the 23-gauge sclerotomy incisions. There are still many important questions regarding the long-term stability and efficacy of the outlined materials and methodology of clot formation and delivery, but further studies have the potential to shape the approach to sclerotomy closure and inform future clinical procedures of wound closure in other ophthalmic surgical procedures.

## Data Availability

Not applicable.

## Abbreviations

PRP: Platelet-rich plasma
IOP: Intraocular pressure
PEG: Polyethylene glycol
